# Liposomes Loaded with Cisplatin and Magnetic Nanoparticles: Physicochemical Characterization, Pharmacokinetics, and In-Vitro Efficacy

**DOI:** 10.3390/molecules23092272

**Published:** 2018-09-06

**Authors:** Alfonso Toro-Cordova, Mario Flores-Cruz, Jaime Santoyo-Salazar, Ernesto Carrillo-Nava, Rafael Jurado, Pavel A. Figueroa-Rodriguez, Pedro Lopez-Sanchez, Luis A. Medina, Patricia Garcia-Lopez

**Affiliations:** 1 Laboratorio de Farmacología, Subdirección de Investigación Básica, Instituto Nacional de Cancerología, 14080 CDMX, Mexico; alfonso.toro@hotmail.com (A.T.-C.); qfb_mariofc@yahoo.com.mx (M.F.-C.); fcojl@yahoo.com (R.J.); 2 Sección de Estudios de Posgrado e Investigación, Escuela Superior de Medicina, Instituto Politécnico Nacional, 11340 CDMX, Mexico; pelosa651018@yahoo.com; 3 Departamento de Física, Centro de Investigacion y de Estudios Avanzados del Instituto Politécnico Nacional, CINVESTAV-IPN, Zacatenco, 07360 CDMX, Mexico; jsantoyo@fis.cinvestav.mx; 4 Laboratorio de Biofisicoquímica, Departamento de Fisicoquímica, Facultad de Química, Universidad Nacional Autónoma de México, 014510 CDMX, Mexico; ernesto.carrillo@unam.mx; 5 Unidad de Investigación Biomédica en Cáncer INCan-UNAM, Instituto Nacional de Cancerología, 14080 CDMX, Mexico; figueroa.pav@gmail.com; 6 Instituto de Física, Universidad Nacional Autónoma de México, 04510 CDMX, Mexico

**Keywords:** cancer, cisplatin, drug delivery, magnetite, liposomes pharmacokinetics

## Abstract

With the aim improving drug delivery, liposomes have been employed as carriers for chemotherapeutics achieving promising results; their co-encapsulation with magnetic nanoparticles is evaluated in this work. The objective of this study was to examine the physicochemical characteristics, the pharmacokinetic behaviour, and the efficacy of pegylated liposomes loaded with cisplatin and magnetic nanoparticles (magnetite) (Cis-MLs). Cis-MLs were prepared by a modified reverse-phase evaporation method. To characterize their physicochemical properties, an evaluation was made of particle size, ζ-potential, phospholipid and cholesterol concentration, phase transition temperature (*T_m_*), the encapsulation efficiency of cisplatin and magnetite, and drug release profiles. Additionally, pharmacokinetic studies were conducted on normal Wistar rats, while apoptosis and the cytotoxic effect were assessed with HeLa cells. We present a method for simultaneously encapsulating cisplatin at the core and also embedding magnetite nanoparticles on the membrane of liposomes with a mean vesicular size of 104.4 ± 11.5 nm and a ζ-potential of −40.5 ± 0.8 mV, affording a stable formulation with a safe pharmacokinetic profile. These liposomes elicited a significant effect on cell viability and triggered apoptosis in HeLa cells.

## 1. Introduction

One of the main disadvantages of cancer chemotherapy is its lack of specificity, which frequently results in dose-dependent damage to normal tissues and therefore affects the tolerability of treatments and the quality of life. Such is the case with cisplatin (*cis*-diamminedichloroplatinum II), one of the most effective and potent anti-neoplastic drugs, which is used to treat more than 50% of human cancers [1,2,3]. The severe adverse effects of this drug include nephrotoxicity, peripheral neuropathy, myelotoxicity, and ototoxicity.

Additionally, the pharmacokinetic behaviour of cisplatin is poor, considering that more than 90% of the drug binds to proteins and thus becomes irreversibly inactivated. Since only a small percentage of the therapeutic dose reaches the site of the tumour [4], efforts are being made to combine cisplatin with a targeted compound to improve delivery. For this purpose, magnetic nanoparticles are now of great interest in targeted therapy for cancers. Drug delivery systems, such as liposomes, are an attractive alternative because the administration of magnetic nanoparticles is hindered by intravenous emulsions.

Liposomes that incorporate magnetic nanoparticles into their structure were first described during the 1980s [5] and have been extensively investigated in recent years due to their potential for specific drug delivery to organs and tissues through controlled magnetic fields [6]. Magnetoliposomes can also be employed in heat-triggered drug release [7,8] and the generation of magnetic hyperthermia; the generation of magnetic hyperthermia is caused by an alternating current (AC) magnetic field (AMF) [9,10,11,12,13]. Hyperthermia is able to yield synergistic effects when combined with some conventional therapies (e.g., radiation and chemotherapy) [14,15,16].

Although encapsulating water-soluble magnetite nanoparticle cores and anticancer drugs in liposomes has been proposed by several researchers [17,18], this strategy is hindered by limitations on the encapsulation efficiency and thermal disruption of the membrane. Regarding encapsulation efficiency, the presence of many nanoparticles represents a large volume, which then reduces the space available for the encapsulation of the drug. Concerning the release of the drug through thermal disruption of the membrane, the high thermal conductivity of water in the vesicle requires heating the entire spherical structure [19]. One option to overcome these limitations is to embed nanoparticles of magnetite in the membrane and encapsulate the cytotoxic drug in the core of liposomes [20,21,22]. In the design of such a system, the challenge is to obtain the combination of a stable and long-circulating liposome in vivo that can be destabilized in a controlled fashion either to facilitate drug release at the target site or to produce hyperthermia. To coordinate drug bioavailability, drug delivery, and therapeutic response, it is essential to study the pharmacokinetics and biodistribution of this kind of liposome.

We herein explored the use of liposome loaded with cisplatin and magnetic nanoparticles. A physicochemical characterization was made of these liposomes, as well as a determination of the pharmacokinetics of this drug delivery system in normal Wistar rats, before proceeding to the evaluation of the therapeutic response in a tumour model. Finally, apoptosis and the cytotoxic effect were assessed in vitro with HeLa cells.

## 2. Results

### 2.1. Physicochemical Characterization

**Figure 1:**
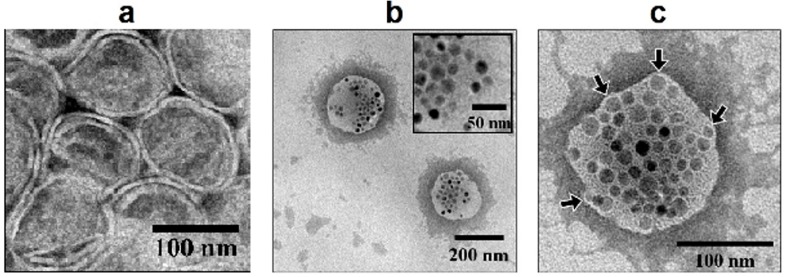
Representative TEM micrographs of (**a**) cisplatin-loaded liposomes (Cis-Ls) and (**b**,**c**) cisplatin-loaded magnetic liposomes (Cis-MLs). All samples were negatively stained with 2% uranyl acetate solution. The inset represents an enlarged view of the magnetite nanoparticles contained in the liposome structure.

The physicochemical parameters of the liposomes loaded with cisplatin and magnetic nanoparticles (Cis-MLs) are summarized in Table 1. The encapsulation efficiency was 8.31 ± 0.4% for cisplatin and 36.0 ± 0.9% for magnetite. The loading capacity was 12.25 mg cisplatin/mol lipid and 10.1 mg Fe^2+^/mol lipid for magnetite. TEM micrographs showed spherical unilamellar vesicles for non-magnetic cisplatin liposomes (Figure 1a) and spherical vesicles with multiple nanoparticles incorporated into their structure for Cis-MLs (Figure 1b). In the Cis-MLs case, there were some deformations in the lipid bilayer (Figure 1c), suggesting that some nanoparticles are located in the membrane while the rest were encapsulated inside the liposome.

**Figure 2:**
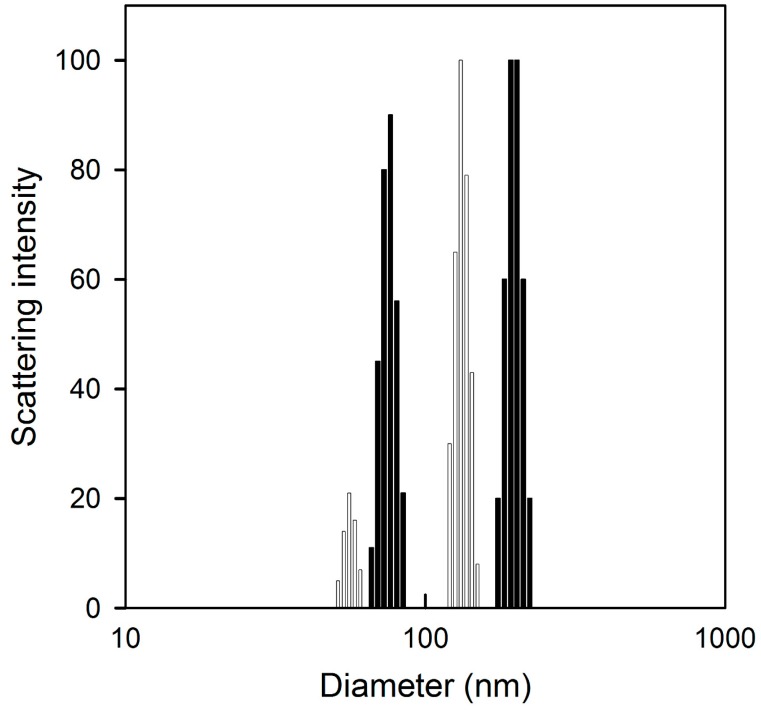
Representative size distribution profiles by dynamic light scattering considering the light intensity of Cis-Ls (grey) and Cis-MLs (black).

The size of the Cis-MLs observed with TEM ranged from 170 to 230 nm, while the hydrodynamic size calculated by dynamic light scattering (DLS) (Figure 2) exhibited a bimodal distribution (range from 70 to 85 and 200 to 315) with peaks at 75 and 250 nm, respectively. In the hydrodynamic size case, the size distributions directly obtained from the intensity of scattered light are biased to higher values as a result of the increase of scattering efficiency with the sixth power of particle size. When a polynomial distribution of sizes is present, the effective diameter calculated by the 90Plus Particle Size Analyzer (Brookhaven Instruments Corporation, Long Island, NY, USA) is an average diameter that is weighted by the intensity of light scattered by each particle. For this reason, the liposome size reported in Table 1 is around 100 nm. It is known that DLS gives an estimation of the hydrodynamic radius of the particle whereas TEM determines the projected area diameter. In DLS, the layer of solvent particles attached to the pegylated liposomes under the influence of Brownian motion affects the estimation of the hydrodynamic diameter. With TEM, the hydration layer is not observed (or considered), and only the projected area of the liposome’s core is used. Both values are important to report; the hydrodynamic size is used to estimate and understand the clearance behaviour of liposomes in blood circulation, while the projected diameter is used to estimate the morphology of liposomes.

**Figure 3:**
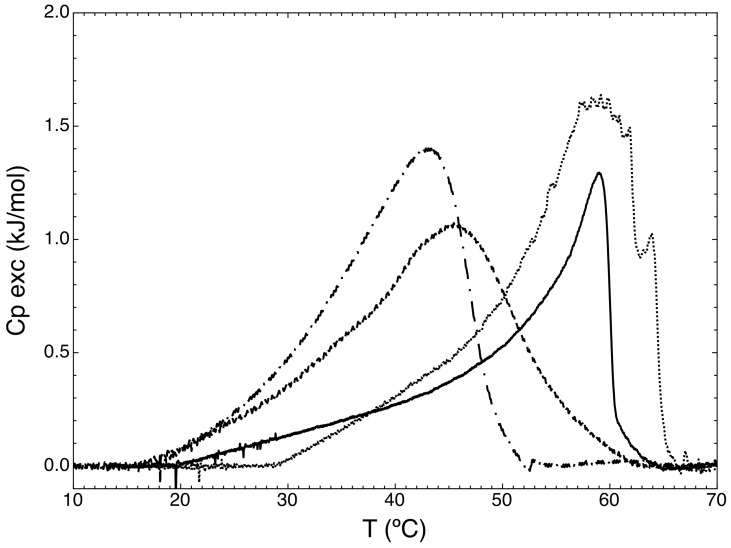
Differential scanning calorimetry of liposome suspensions. Empty liposomes (Ls: —), cisplatin-loaded liposomes (Cis-Ls: ···), blank magnetoliposomes (-- · --), and cisplatin-loaded magnetic liposomes (Cis-MLs: ---).

Calorimetric scanners (DSC) (Figure 3) showed a higher phase transition temperature (*T_m_*) value for cisplatin-loaded liposomes (Cis-Ls) (58.99 °C) than Cis-MLs (45.46 °C). This notorious shift in *T_m_* supports the presence of magnetic nanoparticles at the membrane.

### 2.2. Stability of Cis-MLs

**Figure 4:**
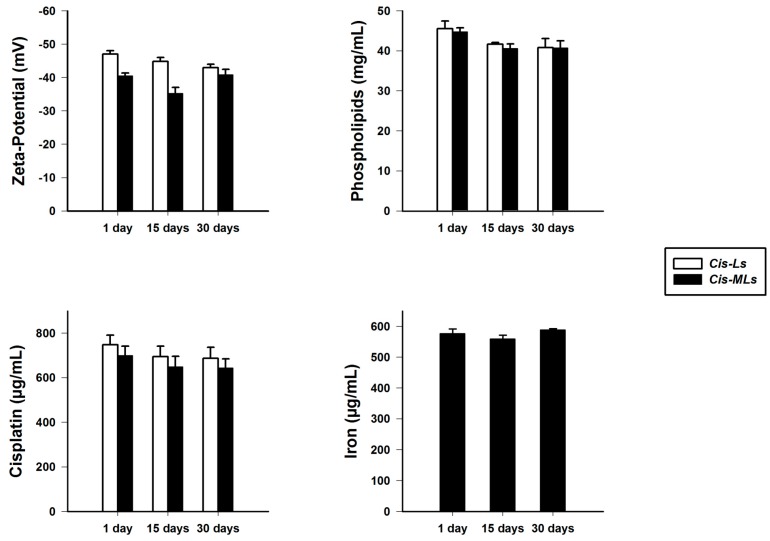
Stability of liposome suspensions in saline solution stored at 4 °C for 15 and 30 days.

**Figure 5:**
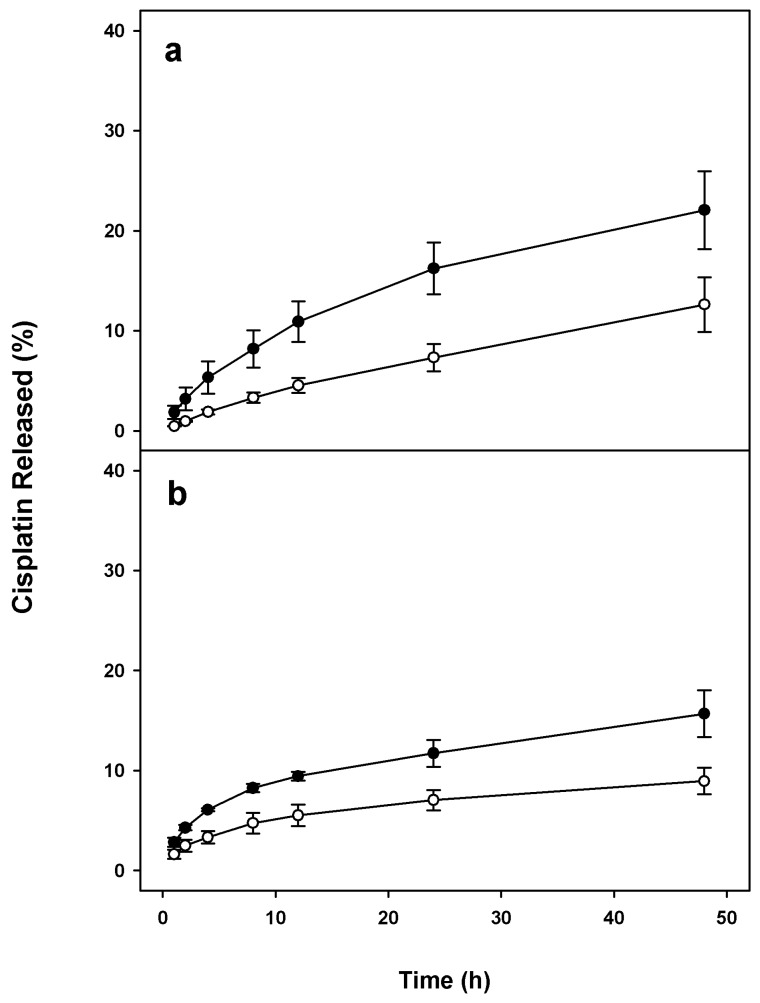
In vitro drug release profiles of cisplatin-loaded liposomes (Cis-Ls, white dots) and cisplatin-loaded magnetic liposomes (Cis-MLs, black dots). (**a**) Percentage of cisplatin released after 48 h of incubation at room temperature in saline solution; (**b**) Percentage of cisplatin released after 48 h of incubation at 37 °C in human serum. Values are expressed as the mean ± SEM (*n* = 3).

The concentration of cisplatin, iron, and phospholipids as well as values of ζ-potential were measured 15 and 30 days after preparation of the liposome suspensions (Figure 4). Drug release profiles of Cis-MLs in saline solution and in human plasma (Figure 5) reveal a slight increase in the release of cisplatin due to the inclusion of magnetic nanoparticles. The maximum release was 22 ± 4% at 48 h in saline solution, being slightly less in human plasma.

### 2.3. Pharmacokinetic and Biodistribution Studies

**Figure 6:**
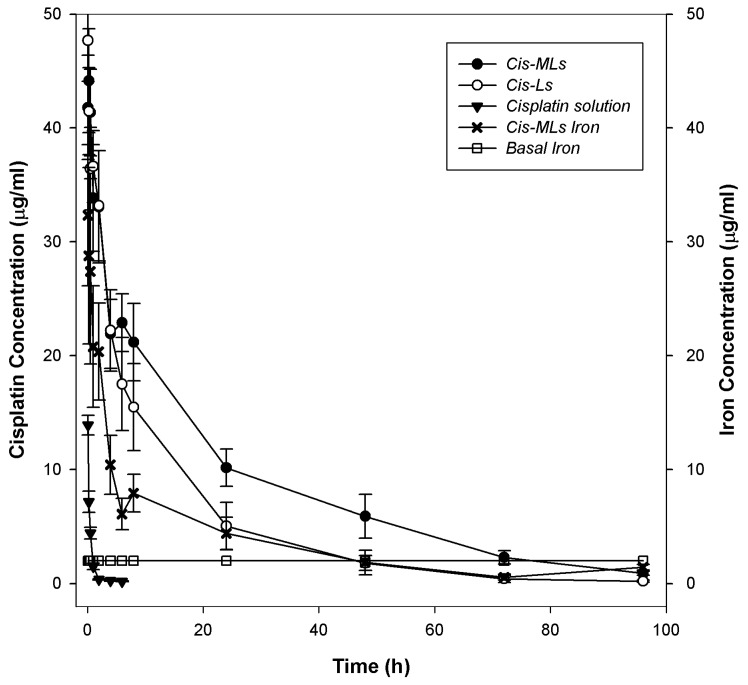
Cisplatin plasma concentrations versus time curves in rats subsequent to intravenous (i.v.) administration of cisplatin solution, cisplatin-loaded liposomes (Cis-Ls), or cisplatin magnetic liposomes (Cis-MLs). Iron plasma concentration versus time curve after i.v. administration of Cis-MLs. The dose injected corresponds to 6 mg/kg cisplatin and 2 mg/kg iron. Values are expressed as the mean ± SEM (*n* = 5–6).

The concentration of cisplatin and iron in plasma is shown in Figure 6. The corresponding pharmacokinetic parameters are listed in Table 2; no significant difference between liposome formulations was detected. It was observed that the half-life time (*t*_1/2_) of Cis-Ls and Cis-MLs in plasma was 5.5-fold longer than that of conventional cisplatin, and clearance of Cis-MLs was reduced more than 80-fold compared to that of conventional cisplatin. Finally, the bioavailability, represented by the area under curve (AUC_0→*t*_), was over 100-fold higher for Cis-MLs and 70-fold higher for Cis-Ls. For iron, it was observed that its clearance is faster than that of cisplatin during the first 24 h and its concentration in plasma was in the order of basal levels after 48 h; the pharmacokinetic parameters are listed in Table 3. It seems that presence of magnetic nanoparticles in the membrane also increases its release from the liposomes. Due to the hydrophobic characteristics of magnetite, it was not possible to compare the pharmacokinetic behaviour of Cis-MLs-Iron to that of free nanoparticles.

**Figure 7:**
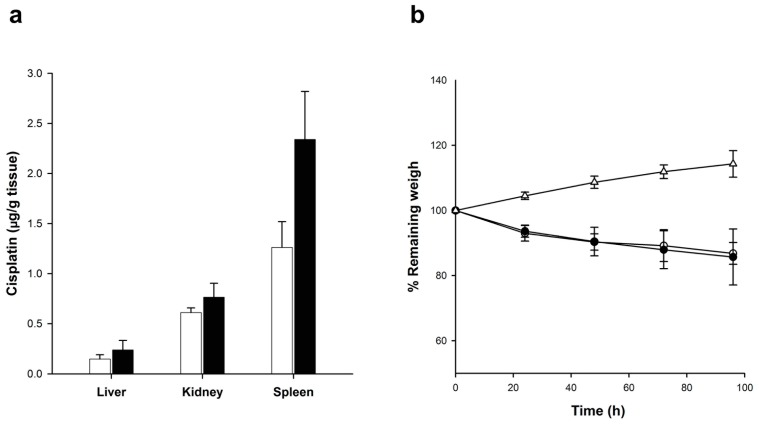
(**a**) Cisplatin accumulation in tissues 96 h after administration of cisplatin-loaded liposomes (Cis-Ls, white bars) or cisplatin-loaded magnetic liposomes (Cis-MLs, black bars); in both cases, the injected dose corresponds to 6 mg/kg cisplatin and 2 mg/kg iron; (**b**) Remaining weight versus time following the administration of Cis-Ls (white dots), Cis-MLs (black dots), and control group (animals without any treatment, open triangles). The animals were weighed before the liposome injection and every 24 h post-administration. Values are expressed as the mean ± SEM (*n* = 5–6).

The concentration of cisplatin in tissues at 96 h post-administration of Cis-MLs is presented in Figure 7. The liver as well as the kidney exhibited similar amounts of cisplatin between treatments (Cis-MLs versus Cis-Ls). However, there was a greater accumulation of cisplatin in the spleen of animals receiving Cis-MLs, from 1.2 ± 0.28 to 2.3 ± 0.47 μg/g of tissue (Figure 7a). The administration of liposomes evidenced a weight loss (around 15% in comparison with the initial weight) in rats treated with Cis-Ls or Cis-MLs (Figure 7b).

### 2.4. In Vitro Study

**Figure 8:**
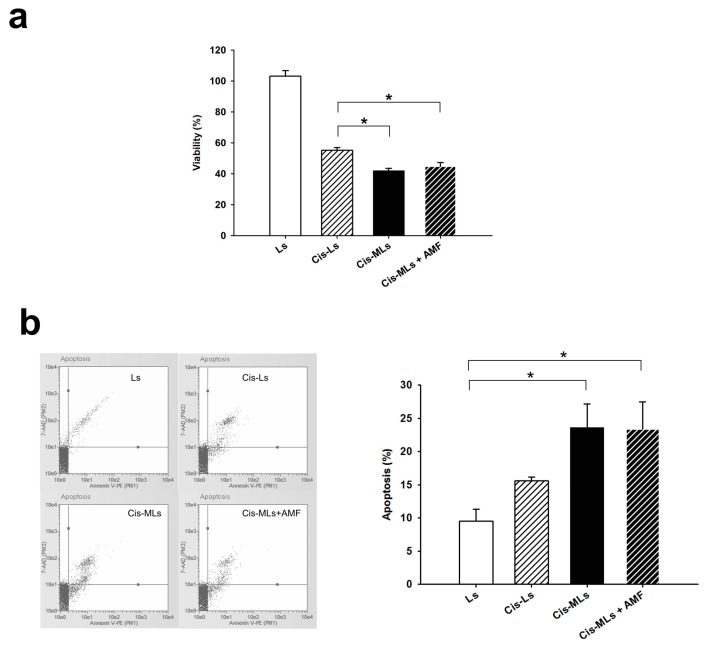
(**a**) Cell viability at 72 h post-treatment with liposomes only (Ls, control), cisplatin-loaded liposomes (Cis-Ls), cisplatin-loaded magnetic liposomes (Cis-MLs), and Cis-MLs plus magnetic hyperthermia treatment with an alternating current magnetic field (AMF) (Cis-MLs + AFM); (**b**) Percentage of apoptotic cells at 72 h after treatment with liposomes only (Ls, control), cisplatin-loaded liposomes (Cis-Ls), cisplatin-loaded magnetic liposomes (Cis-MLs), and Cis-MLs plus magnetic hyperthermia treatment (Cis-MLS + AFM). Values are expressed as the mean ± SEM of three individual experiments. (*) Indicates a significant difference (*p* < 0.05) between the groups.

The 30-min application of an AC magnetic field to cells treated with Ls had no effect on cell viability (data not shown), indicating that the magnetic field itself has no cytotoxic effect. Incubation with Cis-Ls and Cis-MLs significantly (*p* < 0.05) reduced HeLa cell viability compared to the control groups (Ls), but no difference was found between the treatment with Cis-MLs and Cis-MLs plus an AC magnetic field (Figure 8a). Thus, it is suggested that the concentration of magnetite used (40 μM) was not sufficient for the magnetic field to exert a cytotoxic effect. Nevertheless, Cis-Ls produced a less cytotoxic effect than Cis-MLs regardless of the presence of an AC magnetic field. Hence, magnetite itself seems to have affected cell viability. These results are in accordance with the greater percentage of apoptotic cells observed after the Cis-MLs versus Cis-L treatment (Figure 8b).

## 3. Discussion

The reverse-phase evaporation method is reportedly effective for preparing liposomes that enclose water-soluble drugs because the resulting vesicles combine a small particle size with large aqueous volumes. Recently, using this method, multifunctional liposomes for co-encapsulating potential imaging agents with oxaliplatin for treatment by chemo-phototherapy have been developed [23]. By using a modified version of this method, it was possible to achieve the co-encapsulation of both cisplatin and magnetite nanoparticles at the liposome.

Hydrophobic oleic-acid-coated nanoparticles were chosen to avoid interference with the encapsulation of cisplatin. The comparison of Cis-MLs to Cis-Ls reveals that cisplatin entrapment efficiency was not affected by the presence of magnetite. Additionally, the main physicochemical parameters were similar in both formulations (see Table 1), except for the ζ-potential. It was assumed that the increase in the value of the ζ-potential parameter (from −47 to −40 mV) resulted from the presence of the magnetite nanoparticles in the liposome membrane. Despite this rise in ζ-potential, the moderate stability of the suspension avoided flocculation.

The Cis-MLs formed herein had a bimodal distribution of size (ranging from 70 to 315 nm), which is related to the sonication process used to reduce the size of liposome vesicles. A more homogenous size distribution could be attained by extrusion but this process significantly decreases both cisplatin and nanoparticle encapsulation efficiency.

In the current contribution, the stability of Cis-MLs following 48 h of incubation at room temperature was similar to that of other magnetic liposome formulations reported previously [8,24].

The presence and concentration of magnetic nanoparticles in the bilayer of liposomes can produce an important effect on the morphology and the drug release profiles of these vesicles [7]. TEM micrographs (Figure 1) and DSC results (Figure 3) suggest that some of the magnetic nanoparticles were incorporated into the liposome lipid bilayer. The DSC data reveal a significant change in the thermodynamic parameter associated with phase transition. On the other hand, we observed a slight increment in the release of cisplatin from liposome probably due to the presence of 10 nm oleic-acid-coated magnetic nanoparticles in the membrane (Figure 5). This effect is logical considering that the liposome membrane is in the order of 4–6 nm and the presence of bigger nanoparticles (>5 nm) deforms the liposome shape [19,21].

In the pharmacokinetic characterization of a liposomal drug delivery system, it is important to correlate their bioavailability and therapeutic response with the stability and long-circulation behaviour of the vesicles [25,26]. These pharmacological processes strongly depend on the physicochemical characteristics of the carriers. For example, the bioavailability and tissue accumulation of liposomes is modified when their membrane composition or another physicochemical parameter is altered [27]. The current results demonstrate that the plasma pharmacokinetics of liposomes containing cisplatin (Cis-Ls and Cis-MLs) had a significantly higher AUC, a lower rate of clearance, and a smaller volume of distribution (*V_d_*) compared to free cisplatin (Figure 6), indicating a substantial increase in drug bioavailability in both formulations.

It seems the embedding of oleic-acid-coated nanoparticles (10 nm) in the liposome membrane can decisively influence in the permeability and the stability of liposomes (Figure 5 and Figure 6). Indeed, these factors together with the liposome size are expected to be able to affect the pharmacological behaviour of this vesicle. It was observed that the Cis-MLs increases accumulation of cisplatin in the spleen at 96 h (Figure 7a), while concentrations in the liver and kidney were similar for magnetic and non-magnetic cisplatin liposomes. In general, an enhanced liposome uptake in the liver, spleen, and bone marrow is attributed to the phagocytic cells of the reticuloendothelial system (RES) responsible for clearing liposomes from systemic circulation [28,29]. Accumulation in the spleen is common for nanoparticles with a size range between 50 and 200 nm. Liposome uptake from blood circulation is operated by liver Kupffer cells and also by splenic MZ macrophages activated by opsonisation of liposomes. Interestingly, as nanoparticle size increases, Kupffer cell capture decreases and splenic capture is enhanced [30]. The highest accumulation seen in the spleen is in accordance with this behavior as it was observed that the hydrodynamic size of Cis-MLs was up to 315 nm.

Liposomal formulations of antineoplastic drugs have reduced but not eliminated the adverse effects associated with the free drug [31,32]. After the intravenous administration of Cis-Ls or Cis-MLs, the condition of the rats was affected by weight loss (<15%) compared to their initial weight (Figure 7b). However, when compared with the control group (without any treatment), the remaining weights were around 25% at the end of the study. Further toxicity studies are needed to completely guarantee the safety of the cisplatin-loaded magnetic liposomes presently employed.

Several liposomal formulations of cisplatin have been investigated in recent years in order to modify the pharmacokinetics of the drug delivery system. The aim has been to decrease the concentrations of cisplatin in healthy tissues and increase the amount of drug that reaches the tumour. Though they have successfully diminished adverse effects [33,34,35], these formulations have not shown a significant improvement in efficacy or clinical response compared to the standard cisplatin treatments [36]. Non-specific accumulation is suggested as the main cause of therapeutic failure [37,38]. In the present work, an advantage of our system was its slow-release effect. The cisplatin release study and the pharmacokinetic study demonstrated that both agents loaded into liposomes could be sustained for a longer time (prolonged *t*_1/2_) and at a higher bioavailability in the body circulation than the free drug. In other words, given the same systemic dose of the drug, cisplatin and magnetite loaded into a liposomal system could enhance the anti-tumour efficiency.

The amount of encapsulated cisplatin in liposomes is often very limited because of water insolubility and low lipophilicity [39,40,41]. In the current contribution, the encapsulation efficiency of cisplatin was around 10%, which is similar to the values reported by Hirai M et al. [41]. This concentration is enough to perform both in vitro and in vivo studies. The concentration of magnetite in the formulation was considered sufficient for hyperthermia-triggered drug release based on descriptions in the literature [17,42]. More recently, a new method based on an optimization of a prescription with an orthogonal experimental design was reported [43]. In this work, Zhao et al. explore the deliberate loading of magnetic particles in cisplatin-loaded solid lipid nanoparticles to overcome the drawback of low encapsulation efficiency and achieve targeted delivery of cisplatin; this method seems very promising for improving drug delivery in cancer chemotherapy. In future experiments, we could incorporate this experimental strategy using more drugs and improve our system to achieve promising new treatments for cancer.

Under hyperthermia, a rise in temperature (>41 °C) induces cell apoptosis or necrosis due to mechanisms such as protein denaturation, protein folding, and DNA cross-linking [44]. However, no significant effect was presently found on apoptosis or cell viability when Cis-MLs were combined with the application of an AC magnetic field (Figure 8). Regarding magnetic hyperthermia, Néel relaxation, Brownian relaxation, and loss of hysteresis result in thermal energy upon magnetic stimulation. The contribution of each to the specific rate at which magnetic energy is absorbed and converted into thermal energy is strongly dependent on the size, shape, crystalline anisotropy, and degree of aggregation or agglomeration of the nanoparticles [45]. Taking into account the shape and size of the nanoparticles utilized herein (10 nm), Brownian relaxation and the loss of hysteresis do not contribute to the specific absorption rate (SAR), meaning that Néel relaxation is the predominant parameter [46].

Anisotropy refers to the energy barrier associated with rotating the magnetic moment of a magnetic nanoparticle away from its preferred axis, which directly interferes with the decrease in SAR caused by Néel relaxation. In a real system, magnetite nanoparticles, such as those used presently, are seldom perfectly spherical and have a larger crystalline anisotropy that affects the production of thermal energy. The agglomeration of nanoparticles stems from their coating with oleic acid and the reduced space on the membrane in which they are embedded. This parameter probably exerts the greatest influence on the generation of thermal energy. As agglomeration increases, the inter-particle distance decreases, thereby augmenting dipolar interactions that alter the magnetic response [46,47]. These interactions directly modify the Néel relaxation time, the predominant mechanism for the production of hyperthermia in the current system. Hence, agglomeration likely leads to the lack of a significant effect presently found on the viability and on the triggering of apoptosis. Although the application of the AC magnetic field alone did not affect cell viability and apoptosis, it seems that the simple presence of magnetite had a significant effect (Figure 8).

## 4. Materials and Methods

### 4.1. Chemicals

Cisplatin, ferrous ammonium sulphate, ferrozine reagent, nickel chloride, potassium chloride, sodium acetate, sodium chloride, and sodium diethyldithiocarbamate (DDTC) were purchased from Sigma-Aldrich (St. Louis, MO, USA). Uranyl acetate was acquired from SPI Supplies (West Chester, PA, USA). Hydrogenated soybean l-α-phosphatidylcholine (HSPC), 1,2-distearoyl-sn-glycero-3-phosphoethanolamine-*N*-[methoxy(polyethyleneglycol)-2000] (DSPE-mPEG_2000_) and cholesterol were obtained from Avanti Polar Lipids (Birmingham, AL, USA). Oleic-acid-coated iron oxide (Fe_3_O_4_) nanoparticles (magnetite nanoparticles) with a mean hydrodynamic diameter of 10 nm and magnetization superior to 45 emu/g were also purchased from Sigma-Aldrich.

### 4.2. Preparation of Cisplatin-Loaded Magnetic Liposomes (Cis-MLs)

Cis-MLs were elaborated by using a modified version of reverse-phase evaporation [48]. Briefly, lipids (HSPC, cholesterol, and DSPE-mPEG_2000_, 60:35:5 molar ratio) were dissolved in chloroform/methanol (2:1 *v*/*v*) and mixed with 4 mg of magnetite nanoparticles. The mixture was slowly added dropwise into a saturated cisplatin solution in sterile water (8 mg/mL) heated at 65 °C. Once the emulsion was formed, it was placed in a rotatory evaporator to eliminate the organic solvents. The suspension was sonicated for 1 h to reduce and homogenize liposomes size. Non-magnetic cisplatin-liposomes (Cis-Ls) were prepared in the same way but in the absence of magnetite nanoparticles in the lipid solution.

Non-encapsulated cisplatin was removed by dialysis at room temperature (rt) for 4 h in saline solution using with a 3500 Da MWCO membrane (Spectrum Labs Inc., Rancho Dominguez, CA, USA) and non-entrapped magnetite nanoparticles were separated by centrifugation three times at 2500 rpm for 15 min.

### 4.3. Characterization of Cis-MLs

The morphology of the Cis-MLs was evaluated by transmission electron microscopy (TEM) on a JEM 2010 microscope (Jeol Ltd., Tokyo, Japan). Briefly, a drop of the sample was placed on a copper grid coated with carbon film and was allowed to dry for 5 min. Before being viewed under the microscope, the liposome sample was stained with a 2% uranyl acetate solution.

The ζ-potential and diameter of Cis-MLs and Cis-Ls were measured on a 90Plus Particle Size Analyzer (Brookhaven Instruments Corporation, Long Island, NY, USA) at 25 °C. The phospholipid content was quantified by the ammonium ferrothiocyanate method [49]. Briefly, an aliquot of the liposomes (Cis-MLs or Cis-Ls) was dried and mixed with chloroform before adding an equal volume of ammonium ferrothiocyanate. After shaking vigorously, the samples were centrifuged and then the absorbance of the chloroform layer was read at 488 nm in a DU^®^530 spectrophotometer (Beckman Coulter, Woburn, MA, USA). The calibration curve was performed with known concentrations of a solution of HSPC in chloroform.

Cholesterol was evaluated with the commercial kit Advia^®^ Chemistry Chol Reagent (Bayer, Tarrytown, NY, USA). Absorbance was measured at 510 nm and expressed as mg/mL. The amount of encapsulated magnetite was determined by the Ferrozine method [50], which is based on the quantification of ferrous ions. Briefly, an aliquot of the Cis-MLs was mixed with 6 M HCl and incubated for 1 h at 60 °C. Then, the sample was diluted with acetate buffer 5.0 pH containing 0.2 M ascorbic acid and mixed with 20 mM Ferrozine. Subsequent to incubation for 10 min at 37 °C, the sample absorbance was read at 570 nm.

Finally, encapsulated cisplatin was measured by HPLC with a method recently published by our group [51]. Briefly, an aliquot of Cis-MLs was transferred to a centrifuge tube, mixed with acetonitrile, and centrifuged at 10,000 rpm and 4 °C. The supernatant was dried and resuspended in saline solution; nickel chloride was used as internal standard. Cisplatin was derivatized with 10% DDTC and extracted with chloroform. After vigorous mixing and centrifugation, the chloroform layer was injected into a Waters Alliance 2695 HPLC system (Waters Instruments, Milford, MA, USA) fitted with a Waters 2489 UV detector and a Symmetry C18 column (Waters Instruments). The mobile phase consisted of water/methanol/acetonitrile (28:40:32 *v*/*v*/*v*) delivered at 1.8 mL/min. Detection was set at 254 nm.

The entrapment efficiency (EE) and capacity (LC) for magnetite and cisplatin were calculated by the following formulas:

EE (%) = (final amount/beginning amount) × 100(1)

LC (mg/mol) = (mg of cisplatin or iron/mol lipid)(2)

Differential scanning calorimetry (DSC) experiments were performed at 5 to 70 °C utilising a scan rate of 1 °C/min on a VP-DSC calorimeter (Microcal, Northampton, MA, USA). Prior to the scans, all solutions were degassed while being stirred under vacuum. An excess pressure of 3 atm was applied to the cells during scanning. DSC scans were performed from 5 to 70 °C at a scan rate of 1 °C/min. Water versus water scans were performed prior to the liposome scans.

### 4.4. Stability of the Cis-MLs

The concentration of cisplatin, iron, phospholipids, and cholesterol and values of ζ-potential were measured 15 days and 1 month after preparation of the liposome suspensions. They were stored at 4 °C and protected from light.

Cisplatin release from the Cis-MLs was assessed by using Franz diffusion cells assembled with a polycarbonate membrane (pores 0.05 µm) (Millipore Co., County Cork, Ireland) at room temperature in saline solution and at 37 °C in human serum. An aliquot of Cis-MLs was placed into the donor cell compartment and tamped down to the polycarbonate membrane. At predetermined time intervals from 1 to 48 h, the whole receptor phase solution (5–6 mL) was withdrawn and the cisplatin concentration was evaluated by HPLC [49]. At each time point, the percentage of free cisplatin was calculated with the following equation:

% Released = (W_t_/W_0_) × 100 (3)

where W_t_ is the amount in the receptor phase and W_0_ is the initial amount of cisplatin placed in the donor phase.

### 4.5. Pharmacokinetics of Cis-MLs and Cis-Ls

Pharmacokinetic studies were conducted on male Wistar rats (250–300 g; Harlan Laboratories, Coyoacán, Distrito Federal, Mexico), in accordance with the guidelines of the Mexican norm for the handling and care of lab animals (NOM-062-ZOO-1999).

Animals were randomly divided into three groups, receiving (i.v.): (a) 6 mg/kg free cisplatin (non-encapsulated); (b) 6 mg/kg cisplatin liposomes (Cis-Ls); or (c) 6 mg/kg cisplatin magnetoliposomes (Cis-MLs). Rats were anesthetised with isoflurane (Baxter, Estado de Mexico, Mexico) and the corresponding treatment was administered into the jugular vein. Blood samples were collected from the caudal artery at 0, 5, 15, and 30 min and at 1, 2, 4, 6, 8, 24, 48, 72, and 96 h. The rats were sacrificed at 96 h and organs were collected.

Cisplatin in plasma and organs was analysed by HPLC after digestion of the organic material. For the cisplatin solution group, plasma samples were immediately ultrafiltered at 4 °C through Amicon Centrilo cones (10,000 molecular weight-off), and the free cisplatin was determined. Plasma concentrations were plotted against time, and pharmacokinetic parameters, such as the area under the concentration-time curve (AUC), elimination half-life (*t*_1/2_), clearance (*Cl*), volume of distribution (*V_d_*), and plasmatic concentration at time zero (*Cp*_0_) were obtained by using WinNonlin^®^ software (software version, Certara, Princeton, NJ, USA).

Systemic toxicity was evaluated by weight loss and the general condition of animals during the 5 days of each assay. Significant toxicity was considered at 20% of weight loss.

### 4.6. In Vitro Study

The cytotoxic effect of Cis-MLs was examined on 5 × 10^5^ HeLa cells (human cervical carcinoma from ATCC, Manassas, VA, USA). The cells were seeded in 25 cm^2^ flasks and cultivated in Dulbecco’s Modified Eagle Medium (Thermo Fisher Scientific, Waltham, MA, USA) supplemented with 10% foetal calf serum at 37 °C in a 5% CO_2_ atmosphere. Control groups were comprised of cells treated for 24 h with empty liposomes (Ls) and empty Ls plus an AC magnetic field. Treatment groups consisted of cells treated for 24 h with Cis-Ls or Cis-MLs at a concentration of 15 μM cisplatin and cells treated with Cis-MLs plus a 30-min application of an AC magnetic field (Cis-MLs + AMF). The AC magnetic field was created with an experimental setup consisting of a 12-loop cooper coil (1-inch inner diameter) maintained at 37 °C and connected to a radiofrequency (RF) generator operating at a 109 kHz fixed frequency. The calculated magnetic field induction was 17 kA/m.

Following treatments with Ls, Cis-Ls, or Cis-MLs, the cells were trypsinized, suspended in medium, and seeded in a six-well plate at a density of 1 × 10^5^ cells for another 72 h. For the group with the magnetic hyperthermia (Cis-MLs + AFM), the cells were treated in the same manner as the Cis-MLs, except that the trypsinized cells were exposed to an AC magnetic field for 30 min before being seeded in the six-well plates. Cell viability was determined by a crystal violet assay.

Apoptosis was assessed by flow cytometry with a commercial kit (Merck Millipore, Hayward, CA, USA), taking 1 × 10^5^ HeLa cells from each group. They were resuspended in 100 μL PBS and stained with 100 μL of Annexin-V/7-ADD reactive for 20 min in the dark at room temperature. Data were analysed with a Guava EasyCyte cytometer (Millipore^®^) and Guava Nexin software, (guavaSoft^TM^, v.3.1, Hayward, CA, USA).

### 4.7. Statistical Analysis

Statistically significant differences were determined by using one-way analysis of variance (ANOVA) followed by the Bonferroni test to compare data between groups and processed on SPSS Base 20.0 Software (SPSS Inc., Chicago, IL, USA). Data are expressed as the mean ± SEM (standard error of the mean). Significant differences were considered at *p* < 0.05.

## 5. Conclusions

We herein developed a liposomal formulation that successfully co-encapsulates both cisplatin and magnetic nanoparticles; our results suggest that nanoparticles of magnetite were encapsulated in the liposome core but also were embedded in the membrane. It is stable under physiological conditions and during storage. The current pharmacokinetic data reveal that the bioavailability of this liposome formulation was significantly enhanced after intravenous administration. The use of 10 nm oleic-acid-coated magnetic nanoparticles elicited a significant effect of cisplatin on cell viability and apoptosis. However, the generation of thermal energy only caused a minimal increment in cell death. Future research will employ smaller magnetic nanoparticles (<5 nm) to explore their effect on hyperthermia and their synergism with cisplatin to induce greater tumour cell death.

## Figures and Tables

**Table 1:** Physicochemical characterization of liposomes. Main physicochemical parameters measured immediately after preparation.

Parameter	Cis-Ls	Cis-MLs
Particle size (nm)	115.36 ± 4.99	104.43 ± 11.53
ζ-potential (mV)	−47.09 ± 0.95	−40.49 ± 0.87
Lipids (mg/mL)	45.59 ± 1.85	44.79 ± 1.00
Cholesterol (mg/mL)	6.41 ± 0.14	7.29 ± 0.19
Cisplatin (µg/mL)	748.05 ± 43.72	700.17 ± 41.85
Iron (µg/mL)	-	577.29 ± 14.49

Values are expressed as mean ± standard error of the mean (SEM) (*n* = 3). Cis-Ls, cisplatin-loaded liposomes; Cis-MLs, cisplatin-loaded magnetic liposomes.

**Table 2:** Pharmacokinetic parameters of cisplatin after i.v. administration of cisplatin solution, cisplatin liposomes, and Cis-MLs (*n* = 5–6).

Parameter	Cisplatin Solution	Cisplatin Liposomes	Cis-MLs
AUC_0→*t*_ (µg·h/mL·kg)	7.49 ± 0.73 *	519.27 ± 71.07	819.56 ± 140.58
*t*_1/2_ (h)	3.89 ± 2.10 *	22.13 ± 7.14	22.44 ± 1.98
*C_max_* (µg/mL)	21.3 ± 3.61 *	56.2 ± 6.37	45.2 ± 4.47
*Cl* (mL/h)	849.9 ± 102.5 *	12.54 ± 1.9	8.32 ± 1.25
*V_d_* (mL/kg)	313.11 ± 39.6 *	112.7 ± 13.24	139.52 ± 13.94

(*) Indicates a significant difference (*p* < 0.05) between Cisplatin solution versus Cis-Ls and Cis-MLs as determined by analysis of variance followed by a Bonferroni test. (*n* = 5–6). AUC, area under curve.

**Table 3:** Pharmacokinetic parameters of iron after intravenous administration of Cis-MLs (*n* = 6).

Parameter	Cis-MLs
AUC_0→*t*_ (µg·h/mL·kg)	352.84 ± 81.65
*t*_1/2_ (h)	29.48 ± 9.15
*C_max_* (µg/mL)	94.64 ± 59.32
*Cl* (mL/h)	10.12 ± 3.31
*V_d_* (mL/kg)	62.91 ± 18.05
